# A qualitative study to explore the healthcare-seeking experiences of men who have sex with men (MSM) and transgender women (TGW) in Rwanda

**DOI:** 10.1186/s12913-023-09286-x

**Published:** 2023-03-28

**Authors:** Sandra Isano, Tsion Yohannes, Gloria Igihozo, Grace Iliza Ndatinya, Rex Wong

**Affiliations:** 1 Department of Community Health and Social Medicine, University of Global Health Equity, Kigali, Rwanda; 2 Center of Gender Equity, University of Global Health Equity, Kigali, Rwanda; 3 Institute of Global Health, University of Global Health Equity, Kigali, Rwanda; 4 School of Public Health, Yale University, Yale, USA

**Keywords:** MSM, TGW, Key population, HIV, STI, Health care experience

## Abstract

**Background:**

Globally, men who have sex with men (MSM) and transgender women (TGW) encounter many challenging experiences when accessing health services compared to the general population. Stigma, discrimination, and punitive laws against same-sex relationships in some sub-Saharan African countries have made MSM and TGW more prone to depression, suicidal ideation, anxiety disorders, substance abuse, non-communicable diseases, and HIV. None of the prior studies in Rwanda on MSM and TGW had explored their lived experience in accessing health services. Accordingly, this study aimed at exploring the healthcare-seeking experiences of MSM and TGW in Rwanda.

**Methods:**

This study utilized a qualitative research method employing a phenomenological design. Semi-structured in-depth interviews were conducted with 16 MSM and 12 TGW. Participants were recruited via purposive and snowball sampling approaches in five districts in Rwanda.”

**Results:**

Data were analyzed using a thematic analysis approach. Three main themes emerged from the study: (1) The healthcare experiences of MSM and TGW were generally dissatisfactory, (2) MSM and TGW hesitated to seek care unless they were severely ill, (3) MSM and TGW’s perspectives on how to improve their health-seeking behavior.

**Conclusion:**

MSM and TGW in Rwanda continue to face negative experiences within the healthcare delivery settings. These experiences include mistreatment, refusal of care, stigma, and discrimination. Provision of services for MSM and TGW and On-the-job cultural competence training in the care of MSM and TGW patients is needed. Including the same training in the medical and health sciences curriculum is recommended. Furthermore, awareness and sensitization campaigns to improve the understanding of the existence of MSM and TGW and to foster acceptance of gender and sexual diversity in society are necessary.

**Supplementary Information:**

The online version contains supplementary material available at 10.1186/s12913-023-09286-x.

## Background

In many countries, men who have sex with men (MSM) - all men who engage in sexual relations with other men- and transgender women (TGW) - persons whose sex assigned at birth was male but whose gender identity, roles and expression are female [1, 2], often face hostility, social exclusion, discrimination, stigma, irrational fear and even denial of healthcare [3]. In addition, punitive laws against same-sex relationships in many countries have exacerbated this situation. High rates of depression, suicidal ideation, anxiety disorders, substance abuse, HIV, AIDS, and non-communicable diseases are often found among MSM and TGW around the world [4–7].

There are significant barriers to access to healthcare services among MSM and TGW populations [8, 9]. Police harassment, previous challenging healthcare experiences, societal discrimination, medical professionals’ abusive behavior, and refusal of treatment are some of the identified barriers [10]. Such barriers are even more intense in many sub-Saharan African conservative cultures, traditions, and religions, causing MSM and TGW to resort to self-medication, or seeking health advice from their peers or the internet instead of health care professionals [4, 11, 12].

Even in countries where same-sex practices are not criminalized, such as in Rwanda, many MSM and TGW still have similar experiences [13]. Despite healthcare providers’ professional obligation to care for and treat people in an equitable manner, research has shown that prejudice against MSM and TWG remains prevalent [14]. In Rwanda, the fear of being exposed and public humiliation have caused delays in seeking healthcare services, making MSM and TGW more vulnerable to poor health outcomes [13, 15]. Similar situations not only apply to HIV services, but also to cardiovascular, diabetes, and cancer screening services [16, 17].

Although Rwanda has made some progress in terms of protection and promotion of the rights of Lesbian, Gay, Bisexual, Transgender, Intersex (LGBTI+), and gender non-conforming persons from both the legal and policy framework, much work remains to be done. In Rwanda, health services dedicated to MSM and TGW persons are limited and are only being offered by a few community organizations and civil society organizations working on the health, rights, and advocacy of LGBTI + persons. Nearly all these organizations are based in Kigali, the capital city of Rwanda. Additionally, the Rwandan health Sector Strategic Plan 2018–2024, which sets out the national strategic direction for the health sector, does not make any specific mention of LGBTI + persons or their health needs [18]. Although the 2016 Rwanda National Guidelines for Prevention and Management of HIV and STIs state that key populations, including MSM, should routinely undergo non-judgmental STI/HIV risk assessment and client-centered prevention counseling to reduce the HIV and STIs transmission risk, transgender persons are excluded from the National Guidelines for Prevention and Management of HIV and STIs [19]. These guidelines only recognize female sex workers and their clients, men who have sex with men, vulnerable youth (young women 15–24 years), and sero-discordant couples as Key Populations in the context of HIV prevention and response [18, 19].

Prior studies in Rwanda on MSM and TGW mostly focused on their vulnerability to HIV and other STIs [14, 20, 21]. None had explored their lived experience in accessing the health system. Accordingly, this study aimed to generate in-depth information to fill this knowledge gap and to inform the design of appropriate interventions to address the barriers to seeking and accessing healthcare among MSM and TWG in Rwanda.

## Methods

### Study design

A qualitative study using a phenomenological approach was utilized to understand the lived experiences of MSM and TGW when seeking healthcare in Rwanda. This design is one of the most commonly used methodologies in qualitative research to uncover what a given experience means to a group of people who have experienced it [22]. We conducted semi-structured in-depth interviews with 16 MSM and 12 TGW to gain a comprehensive understanding of their experiences. This method was chosen based on recommendations from three Rwandan-led non-governmental organizations with expertise in working with the LGBTI + community in Rwanda. The participants in our study were geographically dispersed across five districts in Rwanda, covering four provinces and the city of Kigali. In-depth interviews were deemed the most appropriate method for data collection due to the dispersed nature of the population, enabling us to collect comprehensive information from a diverse group of participants.

### Recruitment and consent

The recruitment took place between September and October 2021. In this study, we sought to collect data in five target districts that represent all four provinces of Rwanda and the City of Kigali, in order to get information about the diversity of MSM and TGW experiences across the country. Given that MSM and TGW are “hard-to-reach populations” in Rwanda, we purposefully reached out to representatives from three MSM organizations and 2 administrators of TGW organizations (NGOs) who also contacted their members and explained the purposes of the study. The criteria for inclusion were being 18 years or older, identifying as a MSM or a TGW and having sought healthcare services within the last year. Members who agreed to take part in the study were referred to the research team for interviews. Participant recruitment began in Nyarugenge District in the city of Kigali. Each organization representative invited three participants. Among the invited participants, four were TGW, and five were MSM. They were given information about the study aims and recruitment of MSM and TGW and provided with three paper coupons to distribute to potential study MSM and TGW respondents who reside outside of Kigali.

### Data collection

To develop our semi-structured interview guide, we used previous literature as a guide. Our initial step was to conduct a thorough review of the relevant literature on the experiences of MSM and TGW people within Rwanda’s healthcare delivery systems. We analyzed the literature to identify key areas relevant to our study. Additionally, we reviewed questionnaires used in prior studies on the African continent to identify pertinent questions and probes that could inform our research [12, 15]. Using these questionnaires as a foundation, we created an initial draft of our guide. We then refined this draft after receiving feedback from the pilot test. The incorporation of pertinent questions and probes from prior studies on the African continent helped us to create a more comprehensive and contextually relevant guide.

Our final research guide consisted of ten main questions, each supplemented with additional probes designed to gather in-depth information. These questions were centered around the following themes: personal experiences in seeking healthcare, structural barriers to accessing care, interpersonal challenges when interacting with healthcare providers, available resources that facilitate their care, and suggestions to improve health-seeking behavior and health services.

The guide was developed in English and later translated to Kinyarwanda. It was pre-tested with three MSM organization administrators and two TGW organization administrators to ensure its appropriateness for the participants. The feedback received was used to improve the guide’s language and understandability. The guide was then piloted with five MSM and five TGW to identify any issues with language, structure, question flow, and design. Following the piloting, no further changes were made. The involvement of MSM and TGW organizations in the pre-testing and piloting stages helped to improve the guide’s cultural sensitivity and relevance.

To ensure ethical conduct, written consent forms were obtained from all participants before conducting the interviews. Prior to signing the forms, we provided participants with a detailed explanation of the study and answered any questions they had. The interviews were conducted in Kinyarwanda, the local language spoken by the participants, to ensure their full understanding and ease of expression. The interview appointments were arranged based on the participants’ preferred date, time, and location to make the process more convenient for them. All participants received a one-time token of appreciation of 5000 Rwandan Francs, equivalent to roughly 5 US dollars, for their time and participation in the study. However, no incentives were given for the distribution of coupons.

Three people with the necessary skills and training in conducting interviews were selected to carry out the interviews, including two from LGBTI + organizations and the study’s principal investigator. The interviews were conducted in a private and comfortable setting, with each interview lasting approximately 60 min. The principal investigator listened to the interviews and reviewed notes at the end of each data collection day to ensure that saturation was achieved.

To enhance the rigor of the study, the study team engaged in reflexivity practices to critically examine their own biases and preconceived ideas regarding the healthcare-seeking experiences of MSM and TGW. Additionally, the study team met regularly during the data collection period to review the findings and revise the interview guide as necessary.

Data analysis was conducted concurrently with data collection to ensure that the findings aligned with the study objectives and to address any potential challenges. This approach also allowed for monitoring of the data collection process and ensuring that saturation was achieved, which was confirmed after 28 interviews.

### Data analysis

To analyze the collected data, the research team utilized thematic analysis which aimed to uncover patterns and themes that emerged from the data [23]. An inductive approach was adopted, meaning that the themes were derived directly from the data, rather than being influenced by any pre-existing ideas. The “classic” or “basic” method of thematic analysis was employed, which involved five steps: familiarization with the material, coding of transcripts, classification of codes into themes, revision and naming of themes, and interpretation of the results [23–25].

All interviews were recorded and transcribed in Kinyarwanda before being translated into English. To establish an initial codebook, the first four transcripts were reviewed by the full research team, which consisted of four members, two of whom had extensive experience in qualitative methods. The codebook included 11 codes and sub-codes. Teams of two were formed to code the remaining transcripts, and the codebook was refined as new codes emerged.

The team used Dedoose software for data management and analysis [26]. Data visualization was also used to identify patterns and relationships between codes and emerging themes. The team discussed and agreed upon the themes and sub-themes, and representative quotations were included in the findings.

Thematic analysis yielded a comprehensive understanding of the data, highlighting key patterns and themes related to the research question. The inductive approach allowed for a flexible and nuanced interpretation of various aspects of the research topic. The coding framework and data visualization tools ensured that the analysis was systematic and thorough, while the collaboration and consensus among team members helped ensure the reliability and validity of the results. The representative quotations in the findings provided a rich and contextualized understanding of the themes. Overall, the thematic analysis was an effective method for analyzing the data and for uncovering insights related to the research question.

## Results

Twenty-eight in-depth interviews were conducted, with 12 (42.9%) TGW and 16 (57.1%) MSM from all five districts in Rwanda. Their age ranged from 20 to 44 years, with 17 (60.7%) less than 30 years of age. MSM participants’ age ranged from 18-50years, whereas TGW’s age ranged from 20 to 42 years. Many of the participants (n = 20, 71.4%) had completed high school education, and 18 (64.3%) were unemployed at the time of the study (Table 1).

**Table 1:** Demographic characteristics of study participants

Characteristics		N (%)
Sample Size		28
Age (Years)	< 30	17 (61%)
	30–50	11 (39%)
Gender identity/sexual orientation	Trans woman	12 (43%)
	MSM	16 (57%)
Place of residence	Kigali	9 (32%)
	Musanze	3 (11%)
	Muhanga	6 (21%)
	Kayonza	6 (21%)
	Rubavu	4 (14%)
Education	Less than high school	1 (4%)
	High school graduate	20 (71%)
	University level	7 (25%)
Employed	Yes	10 (36%)
	No	18 (64%)

Three major themes emerged from the interviews. The themes are (1) The healthcare experiences of MSM and TGW were generally dissatisfactory, (2) MSM and TGW hesitated to seek care unless they were severely ill, and (3) MSM and TGW’s perspectives on how to improve their health-seeking behavior. All the themes had several subthemes (**see** Table 2 Summary of themes and sub-themes).

### The healthcare experiences of MSM and TGW were generally dissatisfactory

All respondents reported some negative experiences when seeking care at health facilities and generally were dissatisfied with the care they received.

The analysis of the data identified four sub-themes under Theme 1:

(1) Required services were not available in the health facilities, (2) Healthcare providers refused to offer treatment to MSM and TGW, (3) Healthcare providers could not separate sexuality and gender identity from health conditions, and (4) Healthcare providers violated provider-patient privacy and openly mocked MSM and TGW patients.

#### Required services were not available in the health facilities

Respondents stated that many health facilities did not offer the services they needed, particularly for STIs. Some patients were unable to receive the entire service, while others were turned away due to shortages of necessary supplies or a lack of knowledge on the part of healthcare providers. Respondents also reported that some providers were unfamiliar with the diseases commonly experienced by MSM and TGW, leading to suboptimal treatment.

One participant shared a personal experience:

*“Recently, I accompanied my friend to the health facility. He was suffering from anal STI. He had pus coming from the anus. The healthcare provider did not know how to treat that case and told my friend to go to the pharmacy, she said that the pharmacy might have a cream to help with that condition. I was really shocked” (MSM, 36 years old).*

#### Healthcare providers refused to offer treatment to MSM and TGW

MSM and TGW reported dissatisfaction when services were unavailable, but experiences worsened when services were available, yet healthcare providers refused to offer treatment. Some providers would redirect patients to other colleagues, while others ignored MSM and TGW until they left after waiting for extended periods. Additionally, some providers turned them away without treatment due to personal beliefs, perceiving MSM and TGW as sinners or abominations.

A participant shared a distressing experience, stating:

“*Recently I went to the health facility, in fact that was a few months ago but there was no healthcare provider who was ready to receive gays or trans people. I couldn’t receive care. They told me that I should go home and repent because I am a sinner.*” *(TGW, 30 years old)*.

#### Healthcare providers could not separate sexuality and gender identity from health conditions

Some healthcare providers lacked knowledge and awareness regarding homosexuality and gender identity, leading them to view these aspects as illnesses that require treatment. Consequently, healthcare providers focused on addressing these aspects instead of the actual health conditions presented by MSM and TGW patients. In some instances, healthcare providers insinuated that homosexuality was the root cause of the health conditions experienced by the patients. Furthermore, some healthcare providers displayed anger and prejudice towards MSM and TGW, citing societal norms and beliefs.

A participant recounted their experience:

*“They all began telling me that the tumor I had was a result of my anal sex. I was shocked, and I was not convinced by their diagnosis. I mean they really did not treat me; they used that time to convince me to be straight. They even added that I am a curse to my family and society. I was unhappy.” (TGW, 20 years old)*.

#### Healthcare providers violated provider-patient privacy and openly mocked MSM and TGW patients

The professional conduct and ethics of the Rwanda Allied Health Professions Council prohibits violation of patients’ privacy and confidentiality [27]. However, many respondents reported that the healthcare providers did not respect their privacy and would share their personal information with colleagues or other patients, to humiliate them.

One participant shared the following experience:

*Can you imagine, they even shared my result with everyone, luckily, I was HIV negative. I was not satisfied with how I was treated that day”.*

(MSM, 27 years old)

In Summary, Theme 1 demonstrates that most participants had unfavorable experiences when seeking healthcare, and they were dissatisfied with the care they received.

### MSM and TGW generally hesitated to seek care unless they are severely ill

Many respondents were reluctant to seek healthcare unless they were seriously ill, citing negative experiences and stigma as contributing factors. As a result, some used inappropriate or risky treatment methods, lied about their condition, sent someone else to fake a sickness, or self-medicated to avoid abuse.

The analysis of the data identified two sub-themes under Theme 2:

(1) Social exclusion, previous negative experiences with healthcare providers and stigma affected MSM and TGW’s health-seeking behavior, and (2) MSM and TGW resorted to inappropriate and risky treatments, leading to negative health consequences.

#### Social exclusion, previous negative experiences with healthcare providers and stigma affected MSM and TGW’s health seeking behavior

Respondents faced social exclusion from both their communities and healthcare facilities due to their sexual orientation and gender identity. This often led to concealing their identity to healthcare providers and avoiding seeking care until they were severely ill. Previous negative experiences at health facilities, such as denial of services and mistreatment by healthcare providers, further discouraged them from seeking care.

One participant reported this experience:

*I remember one day I went to the health facility for STIs treatment then the healthcare provider who received me he asked me about my sexual partner; I said that I am a gay and I had sex with another man. The healthcare provider looked at me surprised and started asking me questions that conveyed stigma. Some questions were like: “do such people really exist?” Why do you choose to have sex with a man instead of woman? They kept asking many questions instead of serving me. In my mind I said I will never come again to this health center”. (MSM, 30 years old)*

Another participant stated the following:

*“One day when I was severely sick, I think I had an STI. I went to the same health facility which had stigmatized me in the past. While at their gate, I remembered what had happened before, and I decided to stop and went back home. I was afraid of what could happen to me again; I was afraid to meet the same healthcare providers. So, I decided to go in pharmacy and bought medications.” (TGW, 28 years old)*.

#### MSM and TGW resorted to inappropriate and risky treatments, leading to negative health consequences

Many respondents avoided seeking treatment for themselves and instead sent someone else on their behalf to seek treatment due to fear of mistreatment and anticipated negative experiences within the health facility. They preferred seeking medical advice from other MSM and TGW and often resorted to self-medication by buying medicines from pharmacies without proper diagnoses.

One participant shared the following experience:

*“Many healthcare providers mistreat us, and exhibit stigma. I decided to stop going to the health facilities, instead I send a friend to the health center on my behalf when sick or I consult my friends in our MSM community for information about medications I can buy in private pharmacy.” (MSM, 22 years old)*.

Another participant narrated the following:

*“Recently, when I went to the health facility, a nurse I found there looked at me astonished. She started telling me discouraging words, asking me how a handsome man like me could be engaged in homosexuality. She didn’t treat me, and I had to walk to the pharmacy to buy some medicine. I was unhappy and dissatisfied with the services received. On my way back, I lost control to the point where I almost had an accident with the bike I was riding. I was absent minded, wondering why there was so much hate and mistreatment from the healthcare providers. Also, I took the medicine for many days without feeling any relief. I nearly died” (MSM, 22 years old)*.

Overall, in Theme 2, participants reported that their healthcare-seeking behavior was affected by social exclusion, previous negative experiences with healthcare providers, and stigma. Consequently, some participants preferred obtaining medical advice and services from friends, peers, and pharmacies.

### MSM and TGW’s perspectives on how to improve their health seeking behavior

Participants in our study provided suggestions for improving the healthcare system for MSM and TGW, including creating LGBTI+-friendly facilities with designated healthcare providers, training all healthcare providers on MSM and TGW health, advocating for MSM and TGW and reducing stigma through diversity training, and making sexual and reproductive health information and supplies more readily available.

The analysis of the data identified four sub-themes under Theme 3:

(1) Fostering the creation of LGBTI + friendly facilities and having a “focal person” to provide treatment to MSM and Transgender patients, (2) Training all healthcare providers in receiving and providing treatment to MSM and TGW patients, (3) Advocating for MSM and TGW and diversity training for the general public to reduce stigma and (4) Ensuring availability of sexual reproductive health and the right information and material supplies.

#### Fostering the creation of LGBTI + friendly facilities and having a “focal person” to provide treatment to MSM and Transgender patients

Limited availability of LGBTI + friendly facilities, particularly in rural areas, creates challenges for MSM and TGW in accessing health services. Many participants reported having to travel long distances to seek care from trusted providers, which increased the financial burden. To address this, some suggested the creation of dedicated health facilities or having designated focal persons in each facility to provide services to MSM and TGW, which could increase their comfort and trust in using the services.

One MSM participant shared a recommendation for improving healthcare services:

*“My recommendation is to increase knowledge among healthcare providers on how to provide stigma free services because we don’t appreciate the way they treat us. Secondly, where I live there is only one health facility which provides services for the key populations including MSM, and TGW. I recommend that they increase the number of health facilities that can provide such services or else have a focal person at each health facility who could serve us” (MSM, 25 years)*.

Another participant recommended the following:

*“My suggestion is to at least have one trained nurse at the health center who is well informed on LGBTI + health. This would solve many problems. So, we need a focal person to provide service to trans people and MSM (TGW,29 years old)*.

#### Training all healthcare providers in receiving and providing treatment to MSM and TGW patients

Participants emphasized the need for healthcare providers across the country to receive training in handling the health needs of MSM and TGW. Many expressed that the healthcare providers they encountered often lacked the knowledge and skills required to treat MSM and TGW patients and had limited awareness and understanding of the diversity among this population. As a result, participants recommended that healthcare providers in all facilities should be trained on how to better serve and understand the health issues of MSM and TGW patients.

One participant stated the following:

*“Healthcare providers need to be told that we exist. The training [for healthcare* providers*] should focus on reminding them that it is their responsibility to treat people equally, we are all Rwandans! So that we can have access to healthcare services just like any Rwandan, not only relying on only a few health facilities that are specialized to treat LGBTI + I community.” (MSM, 25 years old)*.

#### Advocating for MSM and TGW and diversity training for the general public to reduce stigma

One of the most frequently mentioned recommendations was the importance of advocacy and improved awareness about MSM and TGW to the general public since discrimination and stigma do not only happen within health facilities. Raising awareness about Sexual and Reproductive Health services among MSM and TGW communities was also identified as important. Respondents stressed the government’s responsibility in sensitizing the community and healthcare providers to fight for social justice for MSM and TGW, by addressing their unique needs and challenges in accessing healthcare services. Engaging organizations working on the issue was also recommended. In summary, reducing stigma and discrimination, which adversely affect access to quality healthcare services, requires collaborative efforts of community members, healthcare providers, and the government at various levels.

One participant stated:

*“I wish there could be advocacy so that even policymakers can learn about the issues we are facing. I mean the Rwanda Biomedical Center and even other high level government officials so that they can know that we exist and that our human rights are being violated. We would like them to advocate for us.” (TGW, 25 years old)*

#### Ensuring availability of sexual reproductive health and the right information and material supplies

In addition to promoting awareness and acceptance of MSM and TGW communities among the general public, it is crucial to ensure that these communities are informed about their own health needs and sexualities. MSM and TGW face unique health challenges that require tailored information on sexual and reproductive health, mental healthcare, and LGBTI + health in general. Providing accurate and accessible sexual and reproductive health information, as well as making essential medical supplies such as lubricants, condoms, and pre-exposure prophylaxis (PrEP) available, is critical to improving the health outcomes of MSM and TGW communities.

“*From my own experience, my recommendation would be to ask the Ministry of Health and the Rwanda Biomedical Centre to disseminate the sexual reproductive health and right information to LGBTI + community, the healthcare providers, and all the Rwandans. Condoms, lubricants, and PrEP should also be easy to access. Please help us”MSM, 22 years old)*

Overall, in Theme 3 participants expressed the need for specialized healthcare providers and facilities to cater to the unique health needs and challenges of MSM and TGW. They also emphasized the importance of training programs to increase the knowledge and awareness of healthcare providers on LGBTI + issues.

**Table 2:** Summary of themes and sub-themes on MSM and TGW healthcare seeking experiences

Theme 1: The healthcare experiences of MSM andTGW were generally dissatisfactory	
Sub-theme	Quotations from participants
**(1): Required services were not available in the health facilities**	*“The last time I went to the health center, I needed condoms and lubricants. A nurse who received me told me that lubricants and condoms were not there. She told me to come after two or three weeks to see if they would be available” (TGW, 32 years old)*
**(2): Healthcare providers refused to offer treatment to MSM and TGW**	*“I went to a health facility, the healthcare provider who received me was surprised of seeing transgender women, she went outside and after a few minutes, she came with other colleagues. They surrounded me and it seemed there was something she told them about me. After a few minutes they all left the consultation room. I waited a long time for her to come again, then I felt tired, demoralized and I decided to go without being treated” (MSM, 24 years old)*
**(3): Healthcare providers could not separate sexuality and gender identity from health conditions**	*" And the healthcare providers said : so they have sex with you through your anus, don’t you know that God created the anus to do something different from what you are using it for? God gave you the male genital parts because He knew you are a man! I reply that I was born like that. I did not choose it. I cannot change it, there is nothing else I can do, and I am happy about who I am. They started receiving people who had even arrived after me. They treated me after two hours. It was a bad day”.* *(MSM, 40 years old)*
**(4): Healthcare providers violated provider-patient privacy and openly mocked MSM and TGW patients**	*I went to the health center, but I was not happy with the way they treated me. Among the healthcare providers who were there, some knew\about my sexual orientation. Then, in front of everyone, they started asking me if I had visited the facility because of “the things I do with other men”. Immediately, everyone figured out that I have sex with other men. I was not happy with the way they treated me.* *(TGW, 30 years old)*
**Theme 2: MSM and TGW generally hesitated to seek care unless they are severely ill**	
**Sub-theme**	**Quotations from participants**
**(1): Social exclusion, previous negative experiences with healthcare providers and stigma affected MSM and TGW’s health seeking behavior**	*“When I was suffering from STIs, my colleagues accompanied me to the health facility because I was seriously sick and at that time, I refused to go to the health facility because it was the same place I had been bullied and insulted in the past. But finally, I accepted. When I arrived there, the person who attended to me was a nurse who really made me feel uncomfortable with questions. Moreover, she asked them in front of everyone. I decided to lie to her that I had sex with a woman”. (MSM, 24 years old)”*
**(2): MSM and TGW resorted to inappropriate and risky treatments, leading to negative health consequences.**	*“I don’t usually go to the health facility because I feel uncomfortable sharing that I have sex with another man. I worry that healthcare providers might judge me or think it’s not normal. Instead, I buy medications from the pharmacy or use traditional herbal medicines. My friend told me about a woman who gave me some medicine, but it only helped me for a couple of days. After that, my condition got worse, and it took me a long time to recover. Even now, I still have a burning sensation when I pee.“* *(TGW, 35 years old)*
**Theme 3: MSM and TGW’s perspectives on how to improve their health seeking behavior**	
**Sub-theme**	**Quotations from participants**
**(1): Fostering the creation of LGBTI + friendly facilities and having a “focal person” to provide treatment to MSM and Transgender patients**	*“My wish is to have our own health centers which solely serve the community of MSM, gay and TGW in Rwanda. That’s the only place where I would feel safe.” (TWG, 30 years old)*
**(2): Training all healthcare providers in receiving and providing treatment to MSM and TGW patients**	*“I recommend more training targeting these healthcare providers so that they can learn how to receive us with dignity and to respect us as they do for other people. I also believe that if there was one focal person to receive LGBTI + community at each health center, this would be great”. (TGW, 24 years old).*
**(3): Advocacy for MSM and TGW and diversity training for the general public to reduce stigma are needed**	“*I want to see our government putting more efforts to sensitize society from local authority to the government level about our rights. To end stigma and discrimination, all Rwandans should be aware about our rights and protection as other Rwandans. We too are human beings.” (MSM, 30 years old)*
**(4): Availability of sexual and reproductive health information and material supplies is crucial for better health outcomes among MSM and TGW**	“As a TGW community, I believe it would be beneficial for us to receive training to better understand our sexual orientation and gender identity. This could help us feel more comfortable with who we are and allow us to come out to others. Personally, I’ve struggled with self-acceptance, which made it hard for me to seek health services. However, with the right support and knowledge, I’m hoping more people in our community will be encouraged to seek help when they need it. In addition to this, accessing PEP, PrEP, condoms and lubricants has been a challenge for me and many others in our community. I hope that more support can be provided to ensure that these supplies are available to us, as they are crucial for our sexual health.“ (TGW, 28 years old)

## Discussion

Our study contributes to a limited body of literature on the healthcare-seeking experiences of MSM and TGW in Rwanda [15, 21, 28]. The findings provide unique insights into the challenges faced by MSM and TGW in the Rwandan healthcare system and how stigma and discrimination compound these challenges. Despite being entitled to quality healthcare services like the rest of the population, MSM and TGW in Rwanda continue to face significant barriers to accessing care, including discrimination from healthcare providers, community members, and even their own families. The study also found instances where participants left healthcare facilities untreated due to healthcare providers’ lack of knowledge and skills in treating diseases common to MSM and TGW. To improve healthcare-seeking experiences, participants suggested the implementation of designated facilities for MSM and TGW and training a focal person in treating MSM and TGW at every health facility. A multi-pronged approach, including educational interventions for healthcare providers and the general population, is needed to reduce stigma and discrimination.

In Rwanda, while same-sex relationships are not criminalized, MSM and TGW face similar healthcare-seeking experiences to those in neighboring countries where same-sex acts are illegal, causing many to self-medicate instead of seeking proper care [29–32]. The absence of criminalization does not equate to acceptance, and homophobia and transphobia continue to prevail in Rwanda, resulting in negative health and social outcomes for MSM and TGW. Despite strong cultural and religious beliefs that may not promote tolerance of people with different sexual identities, protecting MSM and TGW through legal mechanisms and social policies can help address health inequities and social injustices [33–36]. Evidence from countries like the United States and South Africa suggests that protective legal mechanisms and social policies could significantly benefit MSM and TGW. To this end, criminalizing homophobic and transphobic acts in Rwanda may be a potential step to protect the rights and health of MSM and TGW [37–40].

Participants in the study commonly expressed the view that healthcare providers lacked the necessary knowledge and skills to treat MSM and TGW. However, this might not necessarily be because healthcare providers lack medical training. It is possible that diversity and cultural competence training for healthcare providers in treating MSM and TGW patients [41] is not included in their basic training curriculum. Providing on-the-job training may help to deliver high-quality, stigma-free services. Collaborating with LGBTI + organizations in Rwanda to develop and deliver training programs could be a solution. Additionally, an online training program may be effective in reducing homophobic attitudes, as seen in Kenya [42]. Hence, piloting a similar training program in Rwanda is worth considering. Our study’s participants emphasized the need for designated healthcare facilities for MSM and TGW to improve their healthcare experience, which aligns with the 2022 WHO guidelines on HIV, viral hepatitis, and STI prevention, diagnosis, treatment, and care for key populations. The guidelines recommend community-led programs to enhance access to health services for key populations. However, caution is necessary in implementing this suggestion in Rwanda, as creating separate clinics for MSM and TGW may exacerbate stigmatization and discrimination. Thus, a careful and deliberate approach is required to ensure that such a proposal does not result in unintended consequences.

Our study’s findings align with those of previous research in Rwanda and the sub-Saharan African region. Isano and colleagues’ 2019 study on HIV post-exposure prophylaxis access barriers among MSM in sub-Saharan Africa found that Rwandan MSM experience significant stigma and discrimination at the family, community, and healthcare levels, which adversely affects their healthcare-seeking behavior [15]. Similarly, in a 2022 study by Gloria et al. in Rwanda that examined healthcare providers’ perspectives on providing HIV prevention and treatment services for key populations, including MSM and commercial sex workers, the findings highlight that both groups face stigma in their communities and healthcare facilities, causing them to be hesitant in seeking medical care [28]. Similar stigma issues leading to limited healthcare access for key populations have been reported in other sub-Saharan African countries, including Uganda and Kenya [3, 15, 28, 29, 43]. Our study’s findings are also in agreement with previous research conducted by Matovu et al. in Uganda, which identified healthcare workers’ limited skills and knowledge in treating MSM [29]. Similarly, a study conducted in Kwazulu-Natal, South Africa, discovered that healthcare providers lacked knowledge in treating TGW patients, resulting in poor relationships between patients and healthcare providers [43]. These results emphasize the need for continuous education and training for healthcare providers to ensure culturally competent and inclusive care for all patients, irrespective of their sexual orientation or gender identity.

This study offers valuable insights into the experiences of MSM and TGW in Rwanda’s healthcare delivery systems. The findings indicate that various stakeholders, including the Ministry of Health, the Rwanda Biomedical Centre, community health organizations, and NGOs, should take measures to reduce stigma and discrimination through educational interventions. Examples of organizations that could contribute to these interventions are the Rwanda Zambia HIV Research Group, Health Development Initiative, Society for Family Health Rwanda, and Alliance for Healthy Communities. Educational interventions should target healthcare professionals and the general population in Rwanda. To ensure that the information is accurate and disseminated effectively, it is essential to involve Rwandan MSM and TGW in the creation and dissemination of educational materials. Future research is necessary to determine the most effective mechanisms for delivering knowledge.

### Strengths and limitations

One strength of this study was the active involvement of representatives from MSM and TGW organizations in the participants’ recruitment process. This collaborative effort allowed the study to reach a diverse sample of MSM and TGW people including those living outside of Kigali City, who may have been challenging to reach otherwise. Additionally, the inclusion of one MSM interviewer and one TGW interviewer as data collectors in our study may have played a crucial role in creating a more comfortable and safer environment for participants during the interview process. Involving community members and organizations ensured that the study was conducted sensitively and inclusively towards MSM and TGW populations, strengthening the quality and validity of the findings. However, the study has some potential limitations. The respondents were initially contacted through MSM and TGW organizations, which usually provide health information to their members, making them more likely to have better access to healthcare services. While efforts were made to collect opinions from different districts, there were still many areas where data could not be collected, limiting the generalizability of the results. The study did not capture the experiences of younger MSM and TGW, and future research should consider this. Finally, participants received a token of appreciation for their time, which may have introduced some biases, although this was not meant to be coercive.

## Conclusion

This study contributes to our understanding of the challenges faced by MSM and TGW in seeking healthcare services in Rwanda. Participants in our sample reported poor experiences with health services, including being denied services due to their sexual orientation or gender identity. These negative experiences have affected their health-seeking behaviors, with many preferring to purchase medicine from pharmacies or receive it from friends instead of seeking services at healthcare facilities. To address these challenges, our participants suggested several strategies, including providing healthcare providers with on-the-job training on cultural competence in caring for MSM and TGW, as well as incorporating such training into medical and health sciences curricula. Our findings underscore the urgent need for program and policy interventions that reduce stigma and discrimination against MSM and TGW in both community and healthcare delivery settings.

## Electronic supplementary material

Below is the link to the electronic supplementary material.

Supplementary Material 1

## Data Availability

The datasets generated and/or analysed during the current study are not publicly available due to privacy and confidentiality considerations but are available from the corresponding author on reasonable request.

## Abbreviations

HIV: Human Immunodeficiency Virus
IDI: In-depth interview
LGBTI+: Lesbian, gay, bisexual, transgender, queer and other gender identities
MSM: Men who have sex with men
MOH: Ministry of Health
NGO: Non-governmental organization
NCDs: Noncommunicable diseases
RBC: Rwanda Biomedical Centre
STI: Sexually transmitted infection
TGW: Transgender Women
