# Supplementary material for: A qualitative study to explore the healthcare-seeking experiences of men who have sex with men (MSM) and transgender women (TGW) in Rwanda
Source: BMC Health Serv Res. 2023 Mar 28;23:291. doi: 10.1186/s12913-023-09286-x (PMC10045920; doi:10.1186/s12913-023-09286-x)
Supplement: Supplementary file 1 — Supplementary Material 1 [file 12913_2023_9286_MOESM1_ESM.docx]

**Tool:** In-depth interview guide : ENGLISH VERSION

**Population:** MSM and Transgender women residing in five districts across all provinces, and Kigali City, in Rwanda

**IN-DEPTH INTERVIEW GUIDE**

1. Explain the interview to the respondent: I am going to ask you a few questions about you, your health, and your experience when seeking healthcare. There are no right or wrong answers. Your thoughts are very important. I will record your answers, and share them with a team of researchers, but no one will know that it is you who gave these responses.

2. Start this interview **after** you have obtained informed consent from the respondent.

3. Start **recording** the interview after you have made introductions.

4. If you do not understand the response, ask the respondent to explain it further, and write down their explanation.

5. Probe for more detail.

6. Be sure to ask if the participant has any questions for you. Reiterate confidentiality. Show appreciation.

**Key Themes :**

- Personal experiences when seeking health care
- Structural barriers in accessing care
- Interpersonal challenges when interacting with healthcare providers
- Available resources that facilitate care
- Suggestions to improve health seeking behavior or health services

**Introduction**

Thank you for the opportunity to speak with you today. The goal of this interview is to collect data on health care seeking experiences of MSM, and Transgender women in Rwanda. The information collected will be used to inform policy and program decisions for improving healthcare delivery and user friendliness of the services provided for MSM, and Transgender women in Rwanda. There are no wrong or right answers. Please be honest and truthful in answering the questions. I would like to record our discussion today. It is likely that I will not be able to write quickly enough to take notes on all of your views and opinions. Recording the discussion will guarantee that all of your responses are available for our research. Do you have any questions before we begin the discussion? Do I have your permission to record our discussion? Thank you. Let’s get started.

**Demographics:** First, I would like to start by gathering some basic information about you. These questions will be used to help me gain a better understanding of your unique situation.

1. What is your birth date? (Ensure that the birth date is provided in dd/mm/yyyy form)
2. What is the highest level of education that you have achieved?
3. What is your current profession or form of employment?
4. **Let’s start by learning more about you. Tell me about yourself, work and your health this year?**

Probe**:** Have you had any recent health concerns? What do you think are the two most important issues for your health now? If you could improve your health right now, what would you do?**.**

1. **Can you describe your last visit to see a doctor (or to a health facility)? Walk me through the experience.**

Probe: Let's start with why you went there ? What happened during the visit? How did you feel about the visit? Were you satisfied with the services ?

1. **Tell me a time when you were sick, and needed care, but you decided not to go to the health facility?**

Probe: Why did you decide not to go? What did you do (alternatives)? Where/who did you get your support from ?

1. **To what extent would you talk with the healthcare providers about your sexual orientation?**

Probe: Barriers faced when accessing health services because of sexual orientation, past experiences, privacy, confidentiality, harassment, education and awareness programs, healthcare providers’ attitudes, cost? Lack of health insurance? Health facility too far away? Transportation issues? Fear? Stigma? Discrimination? income level and personal beliefs?

1. **Some people think it is helpful to discuss one’s sexuality or gender identity with healthcare providers while others think it is not helpful? What are your thoughts on this and how does it influence your healthcare visits?**

Probe: Why helpful/Why unhelpful?

1. **Have you ever withheld info from a healthcare practitioner? Can you describe the experience and why you made that decision?**

1. **Are you aware of any organization / institution that supports MSM/ Transgender women with healthcare services in Rwanda ?**

Probe: How do they help? How did you find out about these organizations? Where are they located

1. **Where do you currently get information about general health related issues?**

Probe: HIV & STI information, access to lubricants, condoms,, mental health information & support

1. **How would you like to see services changed or improved in order to improve your experience when seeking care?**

Probe: What health information would you like to receive pertaining to sexual orientation or practice?How do You Think Healthcare Professionals can Better Support You? What would you recommend to promote healthcare seeking practices of MSM/Transgender women in Rwanda?

1. **Do you have any questions or anything that you’d like to add that we haven’t discussed at this time?**
